# Mast Cells, Pancreatic Stellate Cells, and Telocytes in Chronic Pancreatitis: Ultrastructural Study

**DOI:** 10.3390/ijms262010169

**Published:** 2025-10-19

**Authors:** Irina Chekmareva, Andrey Kostin, Oksana Paklina, Dmitry Kalinin, Dmitry Suraev, Nikolay Karnaukhov, Alexander Alekhnovich, Atim Emaimo John, Viktoria Shishkina, Igor Buchwalow, Markus Tiemann, Dmitrii Atiakshin

**Affiliations:** 1 Federal State Budgetary Institution “National Medical Research Center of Surgery Named after A.V. Vishnevsky” of the Ministry of Health of the Russian Federation, Bolshaya Serpukhovskaya St, 27, 115093 Moscow, Russia; chia236@mail.ru (I.C.); dr.oxanapaklina@mail.ru (O.P.); dmitry.v.kalinin@gmail.com (D.K.); 2 Research Institute of Human Morphology Named After Academician A.P. Avtsyn, Petrovsky National Research Center of Surgery, 3 Tsyurupy St, 117418 Moscow, Russia; 3 Research and Educational Resource Center for Immunophenotyping, Digital Spatial Profiling and Ultrastructural Analysis Innovative Technologies, RUDN University, 6 Miklukho-Maklaya St, 117198 Moscow, Russia; andocrey@mail.ru (A.K.); alekhnovich_av@pfur.ru (A.A.); emaimo_dzhon_a@pfur.ru (A.E.J.); buchwalow@pathologie-hh.de (I.B.); 4 State Budgetary Healthcare Institution “Moscow Clinical Scientific Center Named After A.S. Loginov, Department of Health of the City of Moscow”, Enthusiasts Highway, 86, 111123 Moscow, Russia; nick07@bk.ru; 5 State Budgetary Healthcare Institution “Moscow City Oncological Hospital No. 62, Department of Health of the City of Moscow”, pos. Istra, 27, building 1 to 30, Krasnogorsk Municipal District, 143515 Moscow, Russia; surik195@yandex.ru; 6 Institute of Clinical Morphology and Digital Pathology, Sechenov University, Bolshaya Pirogovskaya Street, Bl. 2/4, 119991 Moscow, Russia; 7 Research Institute of Experimental Biology and Medicine, Burdenko Voronezh State Medical University, 394036 Voronezh, Russia; 4128069@gmail.com; 8 Institute for Hematopathology, Fangdieckstr. 75a, 22547 Hamburg, Germany; mtiemann@hp-hamburg.de

**Keywords:** mast cells, pancreatic stellate cells, telocytes, pancreas, inflammation, fibrosis, pathology, chronic pancreatitis, ultrastructural alterations

## Abstract

Pancreatic inflammation and subsequent fibrosis drive serious disease complications. However, the pathogenesis of this process and the mechanisms underlying excessive extracellular matrix (ECM) deposition remain poorly understood. Our aim was to study intercellular interactions and ultrastructural changes in mast cells, pancreatic stellate cells, and telocytes, as well as in the extracellular matrix in various degrees of pancreatic fibrosis. Histological, immunohistochemical, and electron microscopic (EM) studies were performed on surgical materials from 17 patients. Mapping of fibrosis fields was performed on scanned images using the QuPath software v0.6.0. The IHC study was performed using a panel of antibodies: CD34, CD117, and SMA. Fluorescent IHC was performed using a panel of antibodies: CD34 and CD117. The EM study was performed on ultrathin sections with a thickness of 100–120 nm. The functional activity of mast cells (MCs) increased in pancreatic fibrosis. Most of the MCs were in a degranulation state, with the formation of intercellular contacts. The activation of pancreatic stellate cells (PaSCs), which underwent ultrastructural and functional changes in pancreatic fibrosis that developed as a result of chronic pancreatitis (CP), was noted. Multiple plasmolemma discontinuities, telopode shortenings, and nuclear fragmentations were observed among telocytes (TCs). The presence of MCs in the inflammatory infiltrate, as well as the destruction of TCs with the activation of exosomal transport, plays an important role in the pathogenesis of fibrosis in CP and provides a promising therapeutic target for the treatment of this pathology.

## 1. Introduction

Chronic pancreatitis (CP) is recurrent damage to the pancreatic parenchyma, usually caused by alcohol abuse, as well as autoimmune, genetic, and other factors, leading to exocrine and endocrine dysfunction of the organ. The cardinal characteristics of CP are a triad of signs: fibrosis, atrophy of the acinar tissue, and changes in the ducts [1,2,3,4]. Changes in the ducts depend on the severity of the fibrosis surrounding them. For example, the main duct may have focal constrictions and/or extensions; it may be diffusely affected with uneven dilation and deformation. The ducts usually contain calcareous calculi. Hyperplastic and metaplastic changes are observed in the lining ductal epithelium. In the present context, metaplasia is understood as the so-called acinar–ductal transformation, with the gradual replacement of secretory cells of acinuses by the ductal epithelium, thus forming the so-called tubular complexes. Areas of squamous metaplasia are noted in the ductal system, especially in calculi projections in chronic pancreatitis.

The pathogenesis of CP includes a combination of inflammatory reactions, cellular damage, and fibrosis. It is believed that a cascade of inflammatory reactions leads to the activation of pancreatic stellate cells and their transformation into myofibroblasts, which leads to an increase in the production of extracellular matrix components such as collagen, fibronectin, and laminin. Nevertheless, the role of telocytes as a population of cells that essentially create the 3D structure of the pancreatic parenchyma has not been uncovered, and there is also little research on the role of mast cells and their ultrastructural features in the development of glandular fibrosis. The consequences of the development of pancreatic fibrosis in chronic pancreatitis is the development of exocrine and endocrine organ failure and pain syndrome.

Studies in the last decade suggest that pancreatic fibrosis in CP is the result of excessive deposition of the extracellular matrix in the form of collagen fibers due to the activation of pancreatic stellate cells (PaSCs) [5,6,7,8]. PaSCs are essential for maintaining a normal architecture of the pancreas [9,10]. In their inactive state, PaSCs contain lipid drops with retinol. When PaSCs are activated by inflammatory cells, there is a loss of lipid droplets. PaSCs, similar to myofibroblasts, begin the synthesis of smooth muscle actin and collagen, facilitating the development of pancreatic fibrosis in CP [11]. It has been noted that mast cells (MCs) play a leading role in the activation of PaSCs and in the development of pancreatic fibrosis in CP [12,13,14]. Mast cells are unique regulatory elements of local homeostasis of the local tissue microenvironment, possessing both high sensitivity due to an abundant repertoire of receptors and a high response potential by secreting a wide range of biologically active compounds [15,16,17,18,19,20]. For their communication with other cells, MCs use direct cell-to-cell interactions, the secretion of mediators, and the release of EXOs [21]. MC tools are the three basic classes of mediators—preformed mediators, lipid-derived mediators, and multiple cytokines, chemokines, and growth factors formed after MC stimulation for the requisite modification of physiological responses and immune functions [20,22]. Despite considerable progress, MC biology remains a subject of debate, as does their involvement in a wide spectrum of diseases and chronic allergic/inflammatory conditions, including fibrosis [23]. What remains indisputable is their essential contribution to both innate and adaptive immune responses [23,24,25]. Scientific evidence is accumulating that inter-kingdom communication between human microbiota and MCs is crucial in determining health and disease [26]. The number of MCs in the pancreas in CP has been shown to significantly increase along with their IgE-dependent activation [27,28]. MCs are located in areas of acinar tissue atrophy, which suggests their active involvement in the destruction of acinar tissue and the development of pancreatic fibrosis [14,27].

Apart from MCs, the structural state and functional activity of pancreatic telocytes also play significant roles in the formation of fibrosis [29,30]. Telocytes (TCs) were first described in 2005 [31], while their ultrastructure was described in 2010 [32,33]. A number of researchers show that TC abnormalities are closely associated with many fibrosis-related diseases, such as systemic sclerosis, ulcerative colitis, Crohn’s disease, heart failure, liver fibrosis, endometriosis, and acute salpingitis [34,35,36,37,38,39,40]. However, an unresolved matter remains: whether damage and/or loss of TCs precedes the onset of fibrosis or is a consequence of the fibrotic process [39]. This question remains open. Modern studies, using a model of myocardial infarction and renal fibrosis, confirm that TC transplantation can strengthen angiogenesis, improve structural support in the reconstruction of the TC network in the interstitial compartment, and restore structural and functional support for the activity of stem cell niches, which helps accelerate the restoration of organ function [41,42]. Thus, TCs play a unique role in ensuring functional restoration of the damaged organ and are a promising therapeutic target [43,44]. In this study we analyzed intercellular interactions and ultrastructural changes in cells and the extracellular matrix in patients with various degrees of pancreatic fibrosis.

## 2. Results

Histological changes during CP were uneven and highly varied in different lobules. Periductal and perilobular fibroses were observed in some sections, while intralobular fibrosis was observed in other sections, but the architectonics of the pancreas were partially preserved in the form of lobules, which were replaced by fibrosis to varying degrees. In advanced stages, complete atrophy of the acinar tissue was noted, and accumulations of mononuclears indicated a previously existing destroyed lobule.

**Figure 1:**
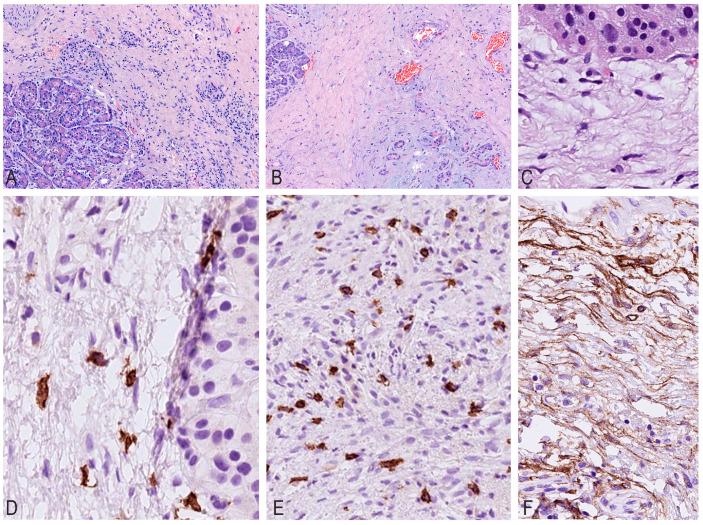
Results of histochemical and immunohistochemical staining. (**A**) Hematoxylin and eosin staining. Moderate fibrosis of the pancreas. (**B**) Hematoxylin and eosin staining. Complete atrophy of the acinar tissue of the pancreas. (**C**) Hematoxylin and eosin staining. The islet of Langerhans is embedded in fibrosis. (**D**,**E**) Expression of CD117 in mast cells in pancreatic fibrosis. (**F**) Increased expression of CD34 in the area of few-cell fibrosis of the pancreas.

It should be noted that, depending on the nature of fibrosis, inflammatory infiltration decreased and its cellular composition changed (Figure 1).

Upon evaluating the sections, it was noted that as fibrous septa and fields formed, the total number of mast cells gradually increased, from 4 to 161 per mm^2^, while the number of lymphocytes and plasmocytes gradually decreased, with mononuclear cells grouped into nodules. The maximum number of mast cells was found in areas with a high density of newly formed capillaries, accompanied by an increased density of other inflammatory cells (Table 1).

The analysis of IHC studies with CD34/SMA antibodies revealed a decrease in expression depending on the maturity of fibrous tissue and hyalinosis, which emphasizes the gradual fibrosis of the parenchyma gland, ranging from “young” cellular fibrosis through mature collagen to cell-free hyaline.

**Figure 2:**
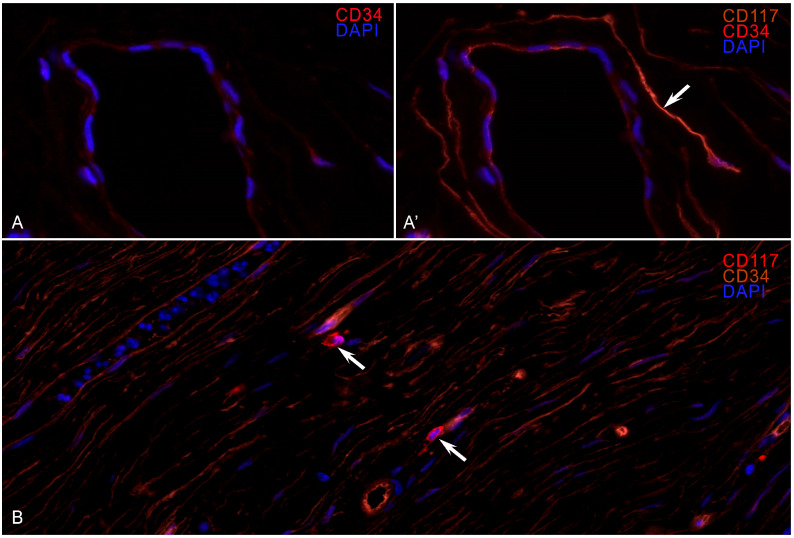
Identification of mast cells and telocytes in the pancreas (fluorescent IHC). (**A**,**A’**) Telocyte staining with CD34 and CD117 (arrow). (**B**) Mast cells are found near the microvasculature.

In the latter, CD34/SMA expression and lympho-plasmocytic infiltration completely disappear, and the number of mast cells decreases to very few per mm^2^. During IHC typing with CD117, the maximum number of mast cells was detected in areas with a high density of newly formed capillaries (Figure 1). Fluorescent IHC revealed colocalization of CD117 and CD34 expressions in the long processes of the TCs (Figure 2).

WSI analysis revealed a violation of the structure of the TC processes, especially in those areas where fibrosis occupied more than 40% of the estimated area. In the areas of hyaline fibrosis, TC processes were not detected. Upon analyzing the fields, it was noted that MCs were localized in groups near the vessels and in the stroma at some distance from the TC telopods. The glow of the markers on the MCs was more intense than the glow of the endothelium and is equivalent to the MCs, but the TC shapes were in the form of a long thin spindle with long telopods and thickenings (podomes), and the MC shape was ovoid or angular with granular cytoplasmic staining.

The morphometric studies performed did not fully reflect the structural and functional state of cells, as well as the nature of changes in the extracellular matrix and intercellular interactions. Further ultrastructural analysis allowed us to elucidate in more detail the state of cellular components involved in pancreatic fibrosis.

### 2.1. Pancreatic Stellate Cells

**Figure 3:**
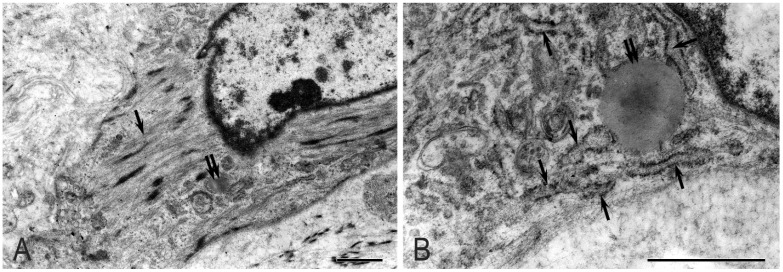
Ultrastructural features of activated pancreatic stellate cells. Electron microscopy. (**A**) Single lipid droplets (double arrow) and microfibrils (black arrow). (**B**) Single lipid droplets (double arrow) and dilated cisterns of the granular endoplasmic reticulum (arrow). Scale: 1 µm.

**Figure 4:**
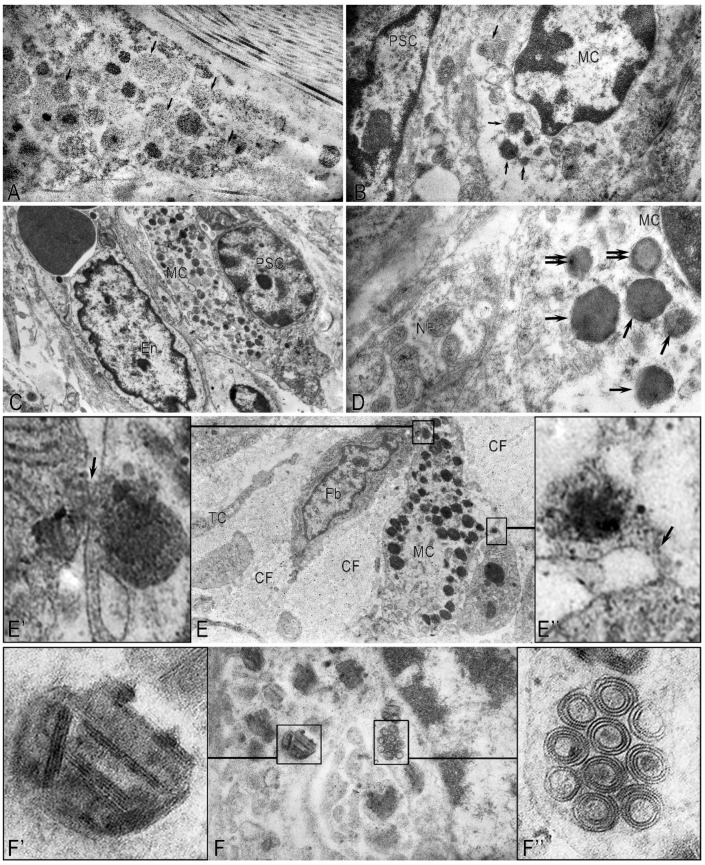
Pancreatic stromal mast cells in chronic pancreatitis. Electron microscopy. (**A**) Mast cells in a state of degranulation. The granules contain varying levels of secretome components, including almost complete absence (arrow). (**B**) Mast cell (**MC**) granules (arrow) in a paracrine location relative to pancreatic stellate cells (**PSCs**). (**C**) Contact between MCs and the endothelial cell (**En**) of the blood vessel and PSCs. (**D**). Contact between the MC and the nerve fiber (**NF**). Contact between the TC and the nerve fiber. Granules in the TC have a uniform (arrow) and uneven (double arrow) filling with the secretome. (**E**) Contact between the MC and two fibroblasts (**Fbs**). Among the collagen fibers (**CFs**) is a telocyte process (**TC**). (**E’**,**E”**) represent enlarged fragments of (**E**). The entry of the MC secretome into the fibroblast (arrow). (**F**) Polymorphism of specific MC granules during degranulation. (**F’**,**F”**) represent enlarged fragments of (**F**).

**Figure 5:**
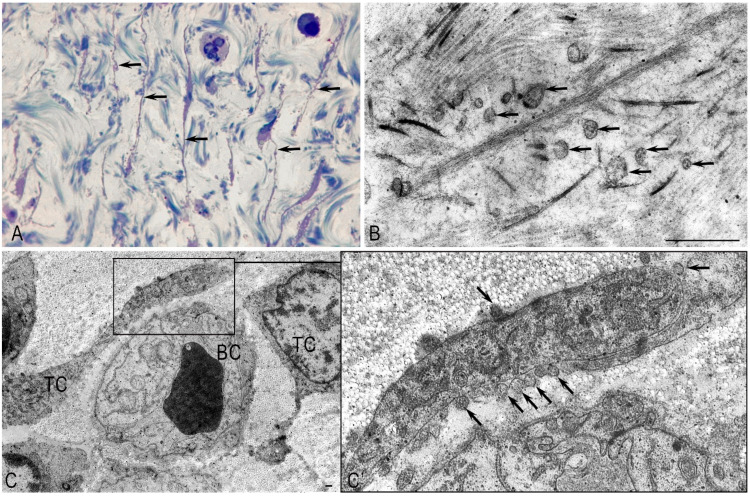
Telocytes in the stroma of the exocrine pancreas in chronic pancreatitis. (**A**) Toluidine blue staining. Long processes of telocytes (arrows) in the fibrously altered stroma of the pancreas. (**B**,**C**) Electron microscopy. (**B**) Microvesicles (arrow) in the stroma among collagen fibrils. (**C**) Telocytes (**TCs**) among collagen microfibrils (**CFs**) surround the blood capillary (**BC**). (**C’**) Enlarged fragment of (**C**). A large number of microvesicles (arrow) are located near the plasma membrane of the telocyte. Scale—500 nm.

In pancreatic fibrosis, due to CP, activation of PaSCs was noted, which underwent ultrastructural and functional changes, including interactions with MCs (Figure 3 and Figure 4). Part of the PaSCs acquired phenotypic features of smooth muscle cells, while other parts acquired signs of fibroblastic differentiation. In the first case, the cytoplasm of cells was filled with bundles of microfibrils with specific dense bodies; however, individual drops of lipids remained in the cytoplasm. In the second case, the cells increased in size and lost lipid droplets (Figure 5A,B). Dilated tubules of the granular cytoplasmic reticulum (GCC) were filled with flaky content and occupied a larger volume of the cytoplasm (Figure 5B).

PaSC activation occurs through exosome, microvesicle, and granulocyte secretions in the intercellular space. Cytokines were damaged by acinocytes and direct intercellular contacts. Thus, activated PaSCs are a cellular source of collagen in pancreatic fibrosis resulting from CP.

### 2.2. Mast Cells

In pancreatic fibrosis, the functional activity of MCs increased. Most of the MCs were in a degranulation state (Figure 4A). The contacts noted by us between degranulated MCs, PaSCs, and endothelial cells with signs of fibroblastic differentiation indicated the direct involvement of MCs in the activation of PaSCs (Figure 4B,C). The EM study of MCs showed that inflammatory cells (eosinophils and lymphocytes) were found in the perineural space of small nerve stems. Contact between degranulating MCs and nerve fibers was noted, which may indicate a neuroimmune interaction, which may further affect the severity of pain in patients with CP (Figure 4D). Contacts between MCs and functionally active fibroblasts were also noted (Figure 4E). Thus, MCs affect the functional activity of PaSCs and fibroblasts and can contribute to the development of fibrosis. MC granules were characterized by ultrastructural polymorphism (Figure 4F). We found a non-homogeneous mixture of granules consisting of large particles and scrolls, dense parallel threads, and fine-grained materials.

### 2.3. Telocytes

Normally, in the unaltered pancreas, the TC processes establish close spatial relationships with the structural components of the pancreatic stroma, forming a complex three-dimensional network within the stromal compartment. TCs were observed in the inter-acinar space, near the acinuses, near the ducts, or in capillaries (Figure 5). It is possible that TCs contribute to their contractility. Telopodes were embedded between collagen fibrils and/or thin collagen fibers (Figure 5A). A large number of microvesicles and multivesicular bodies were observed near the telopodes and the stroma’s structural elements (Figure 5B). A large number of microvesicles were found in the telopods, and caveoles were detected on the surface of the TC processes near the podom (Figure 5C,C’).

The interaction between telocytes and pancreatic cells occurs through direct contacts between microvesicles, exosomes, and probably numerous caveoles. The bilateral interaction process with cells emphasizes the regulatory function of telocytes in the pancreas. Thus, TCs build a complex network structure with various types of intercellular communication, including two-way signal transmission, both to the structures of the pancreatic stromal compartment and from the structures to the TCs. In CP, long TC processes were observed in the fibrous stroma (Figure 5A). The number of TCs decreased as a result of their destruction and necrosis. Extracellular vesicles (exosomes, microvesicles, and multivesicular corpuscles) were found in large numbers among the collagen fibers (Figure 5B) or near the collagen-encased telopods (Figure 5C,C’). A large number of collagen fibers restricted the free flow of telopods in the extracellular matrix of the pancreas and affected the formation of 3D structures by telocytes. Multiple ruptures of the plasmolemma, the shortening of telopods, and fragmentation of the nucleus are characteristic ultrastructural signs of destructive changes in the TCs, which are practically “enclosed” in collagen fibrils during CP.

### 2.4. Features of the Ultrastructure of the Intercellular Matrix in CP

**Figure 6:**
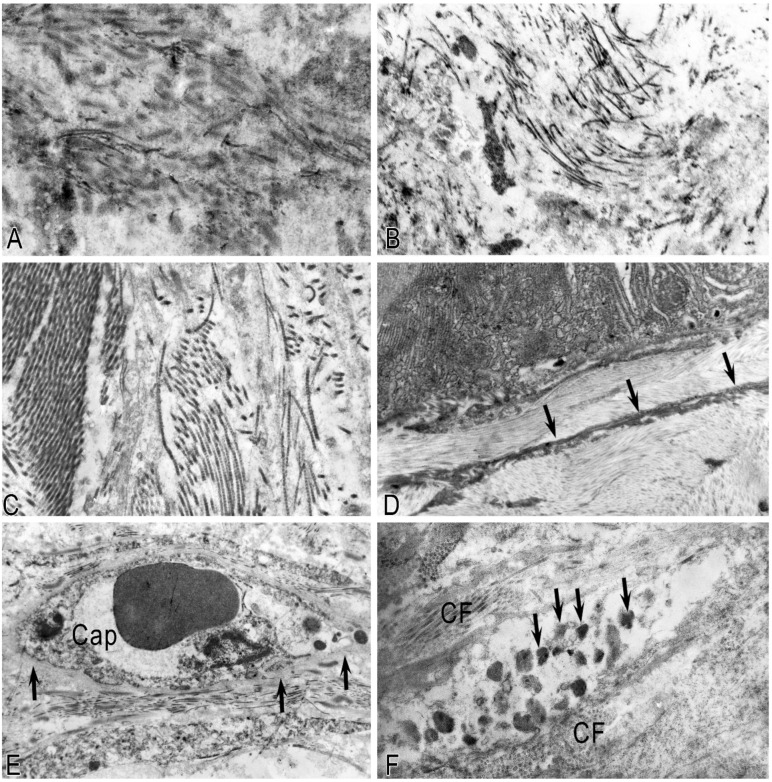
Features of the structure of the intercellular matrix in chronic pancreatitis with severe fibrosis. (**A**) Fibrinoid swelling of collagen fibrils. (**B**) Fragmentation and heterogeneity of collagen fibrils. (**C**) Hyaline-like structures among collagen fibrils. (**D**) The destructively altered TS process (arrows) is embedded in the collagen layer. (**E**) A destroyed capillary (**Cap**) in the fibrously altered stroma with areas of hyalinosis (arrow). (**F**) An autonomous fragment of a mast cell among collagen fibers (**CFs**). Most secretory granules lose their round shape (arrow).

In the early stages of CP, swelling, pronounced loosening, fragmentation of collagen fibrils, and the formation of hyaline-like structures represented by fibrillar proteins were noted. With the progression of fibrosis of the pancreatic stroma, consolidation of collagen fibers, the appearance of hyalinosis sites, and an increase in destructive changes in the pancreatic TCs and blood vessels in the fibrous-altered pancreatic stroma were noted (Figure 6).

Activated MCs, releasing biologically active substances, affect not only the functional activity of cells but also participate in the remodeling of the extracellular stroma. In pancreatic fibrosis, the MCs, like other cells, are immured in collagen fibers. Many MCs were in a state of degradation, and specific granules partially or completely lost their electron density. As a result of the desynchronization of the processes of collagen synthesis and destruction, an excessive amount of collagen is synthesized.

Thus, with severe fibrosis of the pancreatic stroma, the extracellular matrix completely changes, while the possibility of maintaining a spatial 3D network inside the stromal compartment disappears, together with the possibility of cell interaction using microvesicular and exosomal transport.

## 3. Discussion

In the modern literature, when describing the pathogenesis of pancreatic fibrosis in CP, the key role is assigned to PaSCs. Numerous studies show that activated PaSCs are considered the main source of collagen production in the pancreas during the development of organ fibrosis [45,46].

Zimnoch L et al. discovered a significant increase in the number of degranulated MCs depending on the severity of the pancreatic fibrosis and a parallel increase in the number of activated PaSCs [14]. In our study, the number of MCs also increased parallelly with the fibrosis of parenchyma glands, but it fell sharply in fibrosis with an area above 70%, and in areas with pronounced hyalinosis, MCs were not found.

Under the influence of MC_S_, PaSCs differentiate into fibroblasts and myofibroblasts, which begin to actively produce collagen fibrils and fill the intercellular matrix with them. MCs at this stage of the disease can contribute to the fibrotic process, stimulating fibroblasts with profibrotic mediators, thereby participating in the pathogenesis of the disease.

Normally, the degradation of synthesized collagen and extracellular matrix components is regulated by matrix metalloproteinases, whose sources could be fibroblasts, macrophages, smooth muscle cells of the vascular wall, and neutrophils. With the help of metalloproteases in tissues, a balance is maintained between the synthesis and destruction of collagen, in the case of desynchronization of these processes, an excessive amount of collagen is synthesized. However, there is currently insufficient information about the biosynthesis and catabolism of collagen and on the causes of desynchronization of these processes in CP.

The role of MCs in the pathogenesis of CP is not limited to its involvement in the inflammatory process and fibrosis. These cells play an important role in the pathogenesis of pain syndrome in CP. People with severe CP pain syndrome have a 3.5-fold increase in the number of MCs compared to patients with a pain-free course of CP [27,47,48,49]. MCs in the degranulation state were often found in the immediate vicinity of nerves, which confirms the functional connection between MCs and nerves [50,51,52,53,54].

Thus, MCs can contribute to the pathogenesis of pain syndrome in CP through degranulation products, which can increase the sensitivity of pancreatic afferent neurons in the ongoing vicious degranulation cycle of MCs.

The mechanism of TC involvement in the progression of fibrosis in CP is not described, although TCs’ role in the development of fibrosis in the skin, kidneys, lungs, intestinal tube, and liver in various chronic diseases is without doubt [29,42,55,56,57,58].

Normally, TCs contribute to maintaining the normal structure of the organ, regulating tissue homeostasis, and forming a complex three-dimensional network within the stromal compartment of the pancreas. These are large cells with dimensions of more than 10 microns with long processes that form complex labyrinths in the form of a framework.

It is known that the regulation of cellular interactions occurs through the production of microvesicles and exosomes. Exosomes and microvesicles migrate through the intercellular matrix channels, carrying information in the form of micro-RNAs. The progressive filling of the intercellular matrix with collagen fibrils prevents the transport of microvesicles and exosomes, blocking the flow of intercellular communication. Due to changes in the intercellular matrix, which is filled and “cemented” with collagen fibrils, there is a decrease in the number of TCs and an increase in activated PaSCs [14]. A decrease in the amount of TCs in the pancreas leads to a change in the three-dimensional organization of the organ, which is accompanied by a decrease in intercellular interactions and an increase in pancreatic fibrosis. In turn, increasing pancreatic fibrosis leads to the atrophy of cellular structures and macroscopic deformity of the organ, which we observe in CP, especially with a predominant lesion of the pancreatic head.

## 4. Materials and Methods

### 4.1. Case Selection

Histological, immunohistochemical (IHC), and electron microscopic (EM) studies were performed on the surgical materials of 17 patients after various surgical resections for CP or chronic calculous pancreatitis with the presence of post-necrotic pseudocysts. The patients included 14 men and 3 women, 26–64 years of age, and the average age of the patients was 45 years. The control group consisted of 6 samples of unchanged pancreatic tissue after surgical resection of tumors without macro- and microscopic signs of pathology in these areas. This study was conducted in accordance with the principles of the World Medical Association Declaration of Helsinki “Ethical Principles for Medical Research Involving Human Subjects” and approved by the Institutional Review Board of Vishnevsky Surgical Institution (approval protocol no. 007/18, 2 October 2018). Informed consent was obtained from all enrolled participants.

### 4.2. Tissue Probe Staining

The tissue probes left after the routine diagnostic procedure were fixed in buffered 4% formaldehyde and embedded in paraffin. Paraffin tissue sections (5 and 2 µm thick for histochemical and immunohistochemical staining, respectively) were deparaffinized with xylene and rehydrated with graded ethanol according to a standard procedure [59]. Tissue probes of approximately 1 mm^3^ were fixed in 2.5% glutaraldehyde and 1% osmium tetroxide solutions and analyzed using electron microscopy [59].

### 4.3. Immunohistochemistry and Histochemistry

For the immunohistochemical assay, we subjected deparaffinized sections to antigen retrieval by heating the sections in a steamer with the R-UNIVERSAL Epitope Recovery Buffer (Aptum Biologics Ltd., Southampton, UK) at 95C for 30 min [60]. After antigen retrieval and, when required, endogenous peroxidase quenching, the sections were incubated with primary antibodies. IHC studies were performed with antibodies specific to fibroblasts, myofibroblasts, vascular smooth muscle cells (CD34—clone QBend10, Cell Marque, USA and SMA—clone 1A4, Dako, Glostrup, Denmark), and mast cells (CD117—clone EP10, Ventana Medical Systems Inc., Tucson, Arizona, USA). Secondary goat anti-mouse or anti-rabbit antibodies (AmpliStain anti-Mouse 1-Step HRP or AmpliStain anti-Rabbit 1-Step HRP [SDT GmbH, Baesweiler, Germany]) were applied for monoplex immunohistochemical detection of molecular targets with the DAB Peroxidase Substrate Kit (Vector Laboratories, Burlingame, CA, USA) according to the instructions.

For fluorescent IHC, 13 cases out of 17 with the most demonstrative parenchymal changes were selected, where various degrees of fibrosis, mast cells, and hyalinosis fields were viewed. The panel for the fluorescent IHC study included unconjugated labeled primary antibodies to CD117 (Dako, Glostrup, Denmark) and CD34 (Dako, Glostrup, Denmark). To visualize the primary antibodies, secondary antibodies conjugated with fluorescent dyes were used: iFluor 647 (Huabio, Hangzhou, China) and iFluor 594 (Huabio, Hangzhou, China).

Histochemical staining with Mayer’s hematoxylin and eosin was performed according to the manufacturer’s instructions.

### 4.4. Controls

Control incubations were performed by omitting primary antibodies or substituting primary antibodies with the same IgG species (Dianova, Hamburg, Germany) at the same final concentration as the primary antibodies. The exclusion of either the primary or secondary antibody from the immunohistochemical reaction and the substitution of primary antibodies with the corresponding IgG at the same final concentration resulted in a lack of immunostaining. Specific and selective staining of different cells using primary antibodies from the same species on the same preparation is a sufficient control for immunostaining specificity.

### 4.5. Electron Microscopy

To conduct an EM study, pieces of about 1 mm3 in size were cut out of the surgical materials and then fixed in a 2.5% glutaraldehyde solution and a 1% osmium (VIII) oxide solution. Then the material was dehydrated in alcohols of increasing concentrations, soaked in a mixture of “propylene oxide-araldite resin”, covered with araldite resin, and then placed in a thermostat at 60 °C for 48 h. After analyzing the light-optical samples (section thickness: 1.0–1.5 microns; dyed with toluidine blue), the sites for ultramicrotomy were carefully selected.

Ultrathin sections with a thickness of 100–120 nm were cut out on an LKB ultramicrotome (Bromma, Sweden). Sections were stained with uranyl acetate and lead citrate [59]. An ultrastructural study of samples was performed using a JEM-2100 and JEM 100-CX electron microscope (JEOL, Tokyo, Japan) in transmission mode at an accelerating voltage of 80 KV.

### 4.6. Image Acquisition

Stained tissue sections were observed using a Zeiss Axio Imager.Z2 equipped with a Zeiss alpha Plan-Apochromat objective (100×/1.46 Oil DIC M27). Captured images were processed with the software program “Zen 3.0 Light Microscopy Software Package,” “ZEN Module Bundle Intellesis & Analysis for Light Microscopy,” and “ZEN Module Z Stack Hardware” (Carl Zeiss Vision, Jena, Germany) and submitted with the final revision of the article at 300 DPI. The finished samples were scanned using a PANNORAMIC 250 Flash III DX scanner. Images from the JEM 100-CX microscope were captured on film, and the negatives were analyzed after digitization using an Epson Perfection V850 Pro scanner (Nagano Prefecture, Japan).

### 4.7. Quantitative Analysis

IHC expression of CD34/SMA was evaluated in (% per mm^2^). The number of mast cells was determined in 10 fields of view with a × 40 magnification in the fibrous septae. Mapping of the fibrosis fields was performed on a scanned image of the whole slide (whole-slide image—WSI) using the open access image analysis program QuPath (Open Software for Bioimage Analysis, USA) with the calculation in (% per cm^2^) [61]. Taking into account the size of TCs and their structural organization in tissues, their condition was assessed in fibrous septa and fields per 1 mm^2^ of the studied tissue. Considering the density and wholeness of TCs, a semi-quantitative scale was identified: “+++“—the nuclear part of TCs and long continuous telopode processes; “++“—only long telopods; “+“—fragmented shortened telopode; and “−“—processes are practically not visualized.

### 4.8. Statistical Analysis

Statistical analysis was performed using the SPSS software package (v13.0, IBM, New York, NY, USA). The results are presented as the mean ± the standard error of the mean.

## 5. Conclusions

Taking into account the obtained ultrastructural data, the cascade of pathological changes that lead to the progression of fibrotic changes in the pancreas as a result of CP is as follows: damage (etiological factor); inflammation of the parenchyma; stimulation of PaSCs by granulocytes; changes in the extracellular matrix due to increased collagen synthesis; an increase in the amount of MCs; additional activation of PaSCs by mast cells; a further increase in collagen synthesis; swelling and hyalinization of collagen fibrils; changes in the glycocalyx of cells; impaired intercellular transport of exosomes and microvesicles; violation of the regulatory function of TCs; destruction of TCs; a decrease in the number of TCs; destruction and atrophy of acinar tissue; neuropathy; desynchronization of collagen synthesis; and lysis with the formation of a vicious circle of pathological changes in CP.

We believe that the presence of MCs in inflammatory infiltration, and not only the presence of granulocytes, lymphocytes, and macrophages, as well as the destruction of TCs with impaired transport of exosomes and microvesicles, plays an important role in the pathogenesis of fibrosis in CP and provides a promising therapeutic target in the treatment of this pathology, although the main mechanisms of this process require further study.

## Figures and Tables

**Table 1:** The ratio of fibrosis fields to the number of mast cells and the state of telocytes.

N	Fibrosis % mm^2^ (H-E, QuPATH)	Sma % in Fibrous Septa (IHC)	Cd34 % in Fibrous Septa (IHC)	Number of Mast Cells per 1 mm^2^ (IHC)	State of Telocytes/ Telopods (IF)
1	15	0	95	4	+++
2	17	0	95	5	+++
3	23	0	90	3	++
4	35	3	78	23	++
5	47	5	73	24	+
6	52	12	48	75	+
7	61	15	43	80	-
8	63	12	38	161	-
9	72	30	35	153	-
10	73	26	12	160	-
11	75	27	13	56	-
12	82	32	11	21	-

## Data Availability

All data generated or analyzed during this study are included in this published article. Any additional inquiries may be directed to the corresponding author.

## Abbreviations

MCs	Mast cells
PaSCs	Pancreatic stellate cells
TCs	Telocytes
EM	Electron microscopy
ECM	Extracellular matrix
CP	Chronic pancreatitis
α-SMA	Alpha smooth muscle actin
H-E	Hematoxylin and eosin
IHC	Immunohistochemistry
IF	Immunofluorescence
